# Developing a novel tool to assess the ability to self-administer medication in non-demented in-hospital patients: ABLYMED study protocol

**DOI:** 10.1186/s12877-022-03147-8

**Published:** 2022-05-31

**Authors:** Anneke Maiworm, Robert Langner, Stefan Wilm, Dirk M. Hermann, Helmut Frohnhofen, Janine Gronewold

**Affiliations:** 1 Hospital Pharmacy, University Hospital Düsseldorf, Düsseldorf, Germany; 2 Institute of Systems Neuroscience, University Hospital Düsseldorf, Düsseldorf, Germany; 3 Institute of Neuroscience and Medicine (INM-7: Brain and Behaviour), Research Centre Jülich, Jülich, Germany; 4 Institute of General Practice, Centre for Health and Society (Chs), University Hospital Düsseldorf, Düsseldorf, Germany; 5 Department of Neurology and Center for Translational Neuro- and Behavioral Sciences (C-TNBS), University Hospital Essen, University Duisburg-Essen, Hufelandstr. 55, 45122 Essen, Germany; 6 Department of Orthopedics and Trauma Surgery, University Hospital Düsseldorf, Düsseldorf, Germany; 7 Faculty of Health, Department of Medicine, Geriatrics, University Witten-Herdecke, Witten, Germany

**Keywords:** Self administration, Aged, Treatment adherence and compliance, Geriatric assessment, Real-life behavior

## Abstract

**Background:**

Older people often suffer from multimorbidity resulting in polypharmacy. The correct administration of medication is a crucial factor influencing treatment efficacy. However, tools for evaluating the ability to self-administer different dosage forms of medications are lacking. The objectives of the ABLYMED study are to 1) assess the ability to self-administer different dosage forms of medication in older non-demented in-hospital patients who report autonomous management of medication, 2) identify factors influencing the ability to self-administer medication, and 3) develop a standardized tool to validly assess the ability to self-administer different dosage forms of medications based on the final study results.

**Methods:**

One hundred in-patients from the department of orthopedics and trauma surgery of the University Hospital Düsseldorf  ≥ 70 years of age and regularly taking ≥ 5 different drugs autonomously will be prospectively recruited into the observational cross-sectional single-center ABLYMED study. Patients undergo an interview addressing demographic and clinical information, medication history (which medications are taken since when, in which dose and dosage form, and subjective proficiency of taking these medications), medication adherence, and factors possibly influencing adherence including personality traits and perceived quality of the medication regimen. Quality of the medication regimen is also rated by clinicians according to validated lists. Further, patients receive a comprehensive geriatric assessment including measures of cognition, mobility, and functional status. The ability to self-administer medication is assessed by having patients perform different tasks related to medication self-administration, which are video recorded and rated by different experts. The patients’ self-reported ability will be correlated with the observed performance in the self-administration tasks. Further, factors correlating with the reported and observed ability to self-administer medication will be evaluated using correlation and regression models. Based on the final study results, a novel tool to assess the ability of older patients to self-administer medication will be developed.

**Discussion:**

In addition to guideline-based pharmacotherapy, correct intake of prescribed medication is crucial for optimal therapy of multimorbidity in older people. Tools to validly assess the ability of older patients to self-administer different dosage forms of medications are lacking, but should be included in comprehensive geriatric assessments to secure functional health.

**Trial registration:**

Development of an assessment instrument to evaluate the ability to manage various dosage forms**,** DRKS-ID: DRKS00025788, (date of registration: 07/09/2021).

## Background

More than half of the older population ≥ 65 years of age suffers from chronic diseases and multimorbidity [1]. As a result, polypharmacy also increases with age [2] and is associated with negative clinical outcomes such as adverse drug events, drug interactions, potentially inappropriate prescriptions, functional decline, cognitive impairment, falls, hospitalization, and reduced adherence to medication [3]. Of note, underuse of adequately prescribed drugs is associated with negative clinical outcomes as well [4]. Higher complexity of a medication regimen including different dosage forms (e.g., pills, drops, pens, patches, or inhalers), high frequency of intake, or special intake requirements is associated with lower medication adherence leading to underuse of prescribed drugs [3]. Older individuals with polypharmacy are especially at risk of reduced adherence to a complex medication regimen because of their higher rate of impairment in physical and mental function including reduced vision, hearing, manual dexterity, mobility, and cognitive abilities [5].

Up to now, only the influence of handling errors of inhaler devices on the treatment efficacy of obstructive pulmonary disease has been investigated in more detail in older subjects [6–8]. These studies showed a high prevalence of problems with handling different inhalers [6–8] and a moderate association between the number of attempts required to ensure the correct use of the inhalers and the patients’ manual dexterity and cognitive skills [6]. The ability of geriatric patients to take pills out of different kinds of packaging has been investigated before showing that about 10% could not open blister packages, while more than 50% could not open child-resistant packaging. Failure to open pill packaging was associated with poor vision, cognitive function, and manual dexterity [9]. Additionally, the process of handling different dosage forms of medication has not been evaluated in an objective manner, even though this knowledge is crucial for the success of pharmacotherapy and the planning of support by nursing staff.

Therefore, the aims the ABLYMED study are to 1) assess the ability to self-administer different dosage forms of medication in older non-demented in-hospital patients who report autonomous management of medication, 2) identify factors influencing the ability to self-administer medication, and 3) develop a standardized tool to validly assess the ability to self-administer different dosage forms of medications based on the final study results.

Practical consequences of the ABLYMED study include prescription of nursing support in case of insufficient patient abilities to self-administer medication and adaptation of the medication regimen to the preserved abilities to handle medication in case patients can still partially handle their medication. This will improve patients’ quality of life and help to allocate limited healthcare resources in a reasonable way.

## Methods/design

### Study design and setting

This cross-sectional single-center observational study takes place at the department of orthopedics and trauma surgery at the University Hospital Düsseldorf, Germany. With more than 50,000 in-patients per year and more than 1,200 beds, the University Hospital Düsseldorf is the largest hospital of the state capital of North Rhine-Westphalia, Germany. About 9500 employees work in 29 clinical departments and 30 institutes. The percentage of patients ≥ 70 years of age treated in the department of orthopedics and trauma surgery is about 25%. These elderly patients are being taken care of in a specialized geriatric subdivision.

### Study population

Eligible for inclusion in the study are patients ≥ 70 years of age regularly taking ≥ 5 different drugs autonomously who are admitted to the department of orthopedics and trauma surgery electively or via the emergency department. We intend to include 100 patients as a pilot sample to obtain a sufficiently large dataset for performing correlational and regression analyses. Since there is no previous evidence, it was not possible to perform sample size calculation for our specific research question. The only previous study analyzing a related research question, which observed that the ability of geriatric patients to open pill packaging was significantly associated with vision, cognitive function, and manual dexterity [9], recruited a similar sample size as planned in or study (n = 119). Patients are recruited continuously until the planned sample size is reached. Study participation is voluntary and does not affect further treatment.

#### Inclusion criteria

- In-patient at the department of orthopedics and trauma surgery at the University Hospital Düsseldorf, Germany
- Age ≥ 70 years
- Regularly taking ≥ 5 different drugs autonomously
- Written informed consent

#### Exclusion criteria

- Dementia diagnosis (ICD-10-code F00-F03)
- Legal care
- Insufficient ability to self-administer medication at home (nursing service or help from family/friends)
- Insufficient ability to communicate (language barrier for foreign or migrant patients, severe presbyakusis)
- Poor vision (not able to read information written on medication packaging)
- Agraphia (inability to write)
- Alexia (inability to read)
- Instable clinical condition (intensive care requirement)
- Permanently bedridden
- Palliative condition (life expectancy < 6 months)

### Objectives

#### Main objectives

- Identification of problems to handle medication in different dosage forms
- Identification of factors which are associated with the ability to self-administer medication
- Identification of the need for support in handling medication
- Development of a standardized tool to assess the ability to self-administer medication

#### Secondary objectives

Analysis of the interdependency between

- adherence
- complexity and quality of medication regimen
- observed ability to self-administer medication
- reported ability to self-administer medication
- cognitive abilities and personality traits
- demographic / clinical characteristics

### Study procedures

#### Inclusion of patients

After arrival at the ward of the department of orthopedics and trauma surgery of the University Hospital Düsseldorf, all eligible patients receive an information brochure and are asked to participate in the study by the principal investigator (HF). The patients are given sufficient time to discuss participation with their family, friends, or general practitioner. Further, they can clarify remaining questions with the principal investigator. After informed consent is given and signed by the patient, he/she enters the study. The study was approved by the ethics committee at the faculty of medicine of the Heinrich Heine University Düsseldorf. Patients should use their daily support such as glasses and hearing aids during the study assessments.

#### Measures

##### Patient characteristics

In a face-to-face interview, the following patient characteristics are assessed:

- Sex (male, female, diverse)
- Age (years)
- Body weight (kg)
- Height (cm)
- Handedness (right, left, ambidextrous)
- Housing situation (single-person household, multi-person household, nursing home)
- Marital status (open response format, later classified as single = never married, married = living together with a partner, living apart = married but separated, widowed)
- Education level (open response format, later classified according to International Standard Classification of Education [10])
- Last profession (open response format, later classified according to International Standard Classification of Occupations [11])
- Reason for the current hospitalization (open response format, later classified according to the German modification of the International Classification of Procedures in Medicine [OPS])

##### Medication history

In a face-to-face patient interview with supporting medication lists, the following information regarding current medication are assessed:

- Drug name, active ingredient, Anatomical Therapeutic Chemical (ATC) code, strength
- Dosage form
- Dose
- Duration of prescription
- Self-rated proficiency of taking the respective medication (German primary school grading system from 1 (very practiced/experienced) to 6 (not at all practiced/no experience)

Based on the information of the above-mentioned assessment of medication history, the *quality of pharmacotherapy* is assessed according to the FORTA principle [12], the PRISCUS list [13], and the Beers list [14]. In addition, *complexity of the medication regimen* is assessed according to the German version of the Medication Complexity Score (MRCI-D) [15] and *anticholinergic burden* is assessed according to the anticholinergic burden score for German prescribers (ACB-Score-G) [16]. The medication review is performed by a pharmacist (AM) and a geriatrician (HF).

##### Medication adherence

Medication adherence is assessed via a face-to-face interview using the German version of the Medication Adherence Report Scale (MARS-D) [17]. This questionnaire asks for the frequency of non-adherent behavior using 5 items scored on a 5-point Likert scale (1 = always, 2 = often, 3 = sometimes, 4 = rarely, 5 = never). The total score ranges from 5 to 25, with higher scores indicating better adherence to the prescribed medication.

In addition to the MARS-D, the patients answer questions about factors possibly influencing their adherence behavior. These questions were created by the authors based on own ideas and observations, literature review, and expert surveys. Questions refer to general health, beliefs about taking the right amount of medication, taking helpful medications, not having too many changes in medication, tolerance/side effects of medication, actual/needed support regarding medication intake, failures regarding medication management as well as problems regarding vision, swallowing, recognizing medication, and application of different dosage forms.

##### Medication management skills

In a face-to-face interview, medication management skills are assessed with a modified version of the medication management instrument for deficiencies in the elderly (MedMaIDE) [18]. The MedMaIDE was developed in the USA to assess deficiencies in medication management in community-dwelling older adults with non-medical staff. The MedMaIDE is based on subjective questionnaire data and direct observations of medication-taking behavior. It consists of 20 items covering 3 domains considered important for medication management: 1) what a person knows about the medication he/she is taking (knowledge; 8 items); 2) whether a person knows how to take his/her medication (administration; 6 items); and 3) whether a person knows how to get his/her medication from a doctor or pharmacy (procurement; 6 items). Critical items have been identified within each domain (5 for knowledge and administration each, and 3 for procurement), and are used in calculating the deficiency subscores for each domain and a total deficiency score. If a person is not able to answer an item/perform a task correctly for all prescribed medications, he/she gets a score of 1; thus, higher scores indicate higher deficiency in managing medication. For the ABLYMED study, the MedMaIDE was translated into German by a forward–backward translation procedure performed by a professional translation agency, and only the critical items are used.

##### Video-based evaluation of self-administration of medication

A new method was developed to assess the ability of patients to self-administer medication in different dosage forms in their original packaging (A tablets, B eye-drops, C oral drops, D insulin pen, E patch, F inhaler). The application of each dosage form is explained via an instructional video. Patients perform the tests to assess their ability to self-administer medication in different dosage forms twice (except inhaler test) in a modified order (group 1: ABCDEFEDCBA, group 2: FEDCBAABCDE) to control for sequence effects. Patients’ performance is video recorded, for each dosage form and each trial a separate video film is taken. The different dosage forms should be applied as follows:

- A Tablets
  On the table in front of the patient there are two packs of tablets (one including white tablets in an aluminum blister pack and one including blue tablets in a tablet tube), a pill organizer, and a pill cutter. The patient is asked to put one white tablet each for morning, noon, and evening and one blue tablet for the night into the pill organizer and close the organizer. The patient is then asked to open the pill organizer again, take out the blue tablet, divide it into two halves with the pill cutter, and put one half for the night back into the pill organizer.
- B Eye-drops
  On the table in front of the patient there is a one-dose ophtiole dispenser. The patient is asked to open the ophtiole dispenser.
- C Oral drops
  On the table in front of the patient there is a child-resistant dropper bottle and a teaspoon. The patient is asked to open the dropper bottle and drop 10 drops onto the teaspoon.
- D Insulin pen
  On the table in front of the patient there is an insulin pen. The patient is asked to dial in 12 units and inject it into a ball.
- E Patch
  On the table in front of the patient there is a packed patch. The patient is asked to unpack the patch, peel off the protective liner without touching the sticky part too much, and apply it to the skin of the arm.
- F Inhaler
  If the patient's medication already includes a metered-dose inhaler/dry-powder inhaler, the inhalation technique is evaluated according to the validated scoring system developed by Zambelli-Simões et al. [19]. Thereafter, the inspiratory flow rate is measured for each patient at different resistances using the In-Check Dial G16 (MPV Medical GmbH, Munich, Germany). For this purpose, the patient is asked to breathe in slowly and deeply through the mouthpiece at each resistance setting.

If the patients have difficulties during the self-administration of medication, the experimenter can provide verbal or practical assistance, which is documented.

In 30 patients the assessment is repeated after 5 days to assess test–retest reliability.

Based on the video recordings, 2 independent raters evaluate the patient’s ability to self-administer different dosage forms of medication with a standardized assessment form. In a pilot phase, the form is presented to 15 different raters to evaluate the video recordings of 3 different patients. If the raters encounter problems leading to low interrater agreement, the form will be adapted. For each dosage form and each trial separately, the quality of the video is rated as good, moderate or unusable, with moderate indicating that for example the video starts too late or does not have an optimal focus, and unusable indicating that videos are missing or performance cannot be evaluated at all. Thereafter, each step of the medication administration is scored on a 5-point Likert scale (5 = not possible, meaning practical assistance needed or interruption; 4 = severe difficulties, meaning execution hardly possible or success of therapy at risk; 3 = moderate difficulties, meaning execution significantly slowed down; 2 = mild difficulties, meaning execution slightly slowed down; 1 = no difficulties, meaning correct and fluid execution).

The following steps of medication administration were defined:

- For tablets: taking the white tablets out of the packaging, taking the blue tablet out of the packaging, cutting the blue tablet, putting all tablets correctly into the pill organizer
- For eye-drops: opening the ophtiole dispenser
- For oral drops: opening the dropper bottle, targeting at the teaspoon, correct number of drops on the teaspoon
- For insulin pen: removing the cap from the pen, removing the cap from the needle, dialing in the right dose, injection
- For patch: opening the packing, peeling off protective liner, sticking onto the arm
- For inhaler: see Zambelli-Simões et al. [19]

Finally, a global impression of the ability to self-administer the respective dosage form is scored on a 5-point Likert scale (5 = very bad, 4 = bad, 3 = moderate, 2 = good, 1 = very good).

##### Motor function/functional state

A hand dynamometer (Jamar™ hand dynamometer) is used to measure grip strength of the right and left hand. The maximum handgrip strength correlates well with total muscle mass. A hydraulic pinch gauge (Saehan Model SH 5005) is used to measure tip pinch strength of both hands. In tip pinch, the tip of the index finger and thumb hold the objects and the measure indicates direct strength of the 2 fingers.

To estimate everyday function of the hand and fingers, the patient is asked to open a bottle of water closed by a screw cap, which is rated as successful or not successful.

The grooved pegboard test (Lafayette Instrument®, Model 32,025, https://lafayetteevaluation.com/products/grooved-pegboard) is used to assess manual dexterity and complex visual-motor coordination. The patient is asked to put small pegs into 25 holes on a board as fast as possible. The grooved pegs must be inserted exactly and as soon as possible into appropriately shaped holes of the board. The test is executed with both hands separately, beginning with the dominant hand. For the right hand trial, pegs are placed from the patient’s left to right, for the left-hand trial from right to left. The time the patient needs to perform each trial is recorded. A trial is interrupted after 300 s. In addition, the number of drops and the number of correctly placed pegs per trial is noted. The test is explained to the patient with the help of an instructional video.

As an indicator for appendicular skeletal muscle mass, we determine the circumference of the mid upper arm and the calve at the area of its largest extent [20].

Triceps skinfold thickness is an indicator of subcutaneous fat, which is a proxy for total body fat. Triceps skinfold thickness is measured with a skinfold caliper (Fat Control Inc). With the subject's arm in a relaxed position, the skinfold is picked with thumb and index fingers at the middle of the back of upper arm.

History of falls is assessed in a face-to-face interview and categorized as 1 fall within the previous 12 months, 1 fall within the previous 3 months, or more than 1 fall within the previous 3 months.

The de Morton Mobility Index (DEMMI) [21] was developed to measure mobility in hospitalised older acute medical patients across a broad spectrum of mobility from bed bound to independent mobility. It consists of 15 mobility tasks which vary from easy (such as turning around while lying in bed) to demanding (such as placing the heel of one foot directly in front of the other with eyes closed for 10 s). The raw score is transformed to range from 0 to 100%, with lower scores indicating lower mobility.

The interview-based Katz index [22] assesses dependence in activities of daily living including bathing, dressing, going to the toilet, transferring, continence, and eating. It comprises 6 items, the total score ranges from 0 to 6, with lower scores indicating higher dependence.

The interview-based Barthel index [23] assesses dependence in activities of daily living similar to the Katz index, but in more domains, including help needed with eating, transfer (e.g. from bed to chair), grooming, bathing, toileting, walking, climbing stairs and dressing as well as presence of anal and urinary incontinence. It comprises 10 items, and the total score ranges from 0 to 100, with lower scores indicating higher dependence.

While the Barthel and the Katz index measure dependence in basic activities of daily living, the interview-based IADL scale [24] includes more complex activities of daily living. It comprises 8 items assessing the ability regarding telephone use, shopping, food preparation, housekeeping, laundry, mode of transportation, responsibility for own medications, and handling finances. The total score ranges from 0 to 8, with lower scores indicating higher dependence.

##### Nutritional status

The Mini Nutritional Assessment Short Form (MNA-SF) [25] is used for the interview-based screening for undernutrition in geriatric practice. It consists of 6 items assessing decrease in food intake, weight loss and psychological stress/acute disease during the last 3 months and mobility, neuropsychological problems, and body mass index. The total score ranges from 0 to 14, scores < 11 indicate elevated risk for undernutrition.

##### Cognitive function

The Timed Test of Money Counting [26] is a short test to assess manual dexterity and cognitive capacity. The patient is given a purse with separate pockets for coins and banknotes, which contains a predefined amount of money (9.80 Euros as a 5-Euro note, a 2-Euro coin, 2 1-Euro coins, a 50-cent coin and 3 10-cent coins) and is asked to take out all the money and count it. The time the patient needs to perform this task is measured. If the patient does not report the correct amount of money, the examiner gives the feedback that the answer is not correct and the patient is allowed to try again, with the number of attempts and the problems the patient has with this task being recorded. The time measurement is not interrupted while correcting patients. The Timed Test of Money Counting is interrupted after 3 incorrect answers or after 300 s, and in both cases a duration of 300 s is recorded. Values < 45 s indicate independence, 45–70 s increased risk for dependence, > 70 s increased care needs [26].

The Trail Making Test for older subjects (ZVT-G) [27] is the German version of the Trail Making Test A [28]. The ZVT-G was developed for subjects ≥ 55 years of age and is included in the Nürnberger Alters-Inventar [27]. The patient is asked to connect numbers from 1 to 30 in rising order with a pen. The time the patient needs to perform this task is measured. Mistakes shall be corrected immediately without interrupting time measurement. If the patient makes more than 3 mistakes, the test is repeated. If the patient again makes more than 3 mistakes or after a maximum time of 300 s, the test is interrupted, in both cases a duration of 300 s is recorded.

Drawing the interlocking pentagons taken from the Mini-mental state examination (MMSE) [29] is used to assess visual-spatial skills.

The Clock-drawing test [30] assesses higher-level cognitive abilities including visuospatial skills, integrative functions and abstract thinking. The patient is given a sheet of paper with a pre-drawn circle. The experimenter shows where the top of the page is and gives the instructions to put the numbers on the clock and set the time at 10 past 11. The score ranges from 1 to 6, with higher scores reflecting a greater number of errors and more impairment.

Time estimation is assessed with two tasks: First, the patient is asked to estimate how long one minute is with the following instruction: “Please give me a sign when you think one minute has passed, starting now.” The time (in s) is measured until the patient gives the sign. Thereafter, the patient is asked to estimate his/her current length of hospital stay in days with the following instruction: “How long have you been in hospital?” It is noted whether the response is correct and, if not, how many days the response differs from the actual length of stay.

The interview part assessing factors possibly influencing medication adherence is framed by a task that addresses prospective memory, which is a version of the task used in the UK Biobank project [31] modified to increase difficulty. Prospective memory indicates the ability to carry out future intentions at a specific time or in response to a specific event. At the beginning of the specific interview part, patients receive the following instruction: “At the end of this interview part, we will show you colored symbols and ask you to touch the blue triangle. However, to test your memory, we want you to actually touch the orange square instead”. The patients are shown a sheet with 3 different shapes (triangle, square, circle) presented in 3 different colors each (blue, orange, pink). Their first and final answer, the history of attempts, and whether a hint was given are recorded.

The six-item screener [32] is based on the MMSE [29] and comprises temporal orientation (current day of the week, month, year) and short term memory (ability to recall 3 newly learned words). A cut-off of ≥ 3 errors is applied to identify participants with cognitive impairment [32].

##### Personality traits

Personality of the patients is assessed with the Big-Five-Inventory-10 (BFI-10) [33]. This questionnaire is based on the currently most popular model of human personality, the Big-Five model, which assumes 5 abstract personality dimensions (neuroticism, extraversion, openness, agreeableness, conscientiousness). Each of these dimensions is assessed with 2 items, which are scored on a 5-point Likert scale (1 = disagree strongly‚ 2 = disagree a little‚ 3 = neither agree nor disagree‚ 4 = agree a little‚ 5 = strongly agree). Scores are averaged per dimension and thus range from 1 to 5, with higher scores indicating higher expression of the personality trait.

The study is ongoing, patient recruitment has already started. Statistical analysis will take place in the second half of 2022, publication of the full results is scheduled for the first half of 2023.

### Statistical analysis

Patients’ characteristics regarding all variables assessed will be reported as mean ± standard deviation for continuous normally distributed variables, as median (Q1; Q3) for continuous variables which are not normally distributed, and as numbers (%, valid % when values are missing) for categorical variables.

For the video-based evaluation of self-administration of medication, at first 15 raters will rate a small subset of the patient sample to check whether the proposed standardized evaluation form is feasible, objective, and reliable. The agreement between the 15 raters on the ratings for each patient will be evaluated using Kendall’s W for categorical data (single items) and intraclass correlation coefficient for continuous data (sum scores). If the agreement is sufficiently high (≥ 0.8), the total patient sample will be rated by 2 independent raters and agreement will be evaluated with Cohen’s kappa for categorical data and intraclass correlation coefficient for continuous data. To assess test–retest reliability of the video-based evaluation, 30 patients will perform the assessment again after 5 days, and the 2 raters, who rated the total patient sample, will rate patient performance again. Retest reliability will be calculated using Spearman’s Rho for continuous variables and Chi-square for categorical variables.

To evaluate which factors influence the ability to self-administer medication, correlation and regression analyses will be performed. For continuous scores describing the ability to self-administer medication, Spearman’s Rho will be used for analyzing continuous and ordinal factors and Chi-square for analyzing nominal factors. Curve estimation procedure will be used to check whether a linear regression model fits the data in case of bivariate continuous associations and variables will be transformed if necessary. Uni- and multivariable linear regressions models including interaction terms will be calculated to identify which factors influence the ability to self-administer medication to which degree. If linear associations are not met even with data transformation, non-linear regressions will be performed. Missing data will be excluded from analysis listwise. All statistical analyses will be performed using SPSS 22 for Windows (IBM Corporation, Armonk, NY, USA). All statistical tests will be 2-tailed and *p*-values < 0.05 will be considered significant.

## Discussion

The ABLYMED study aims to develop a feasible, objective, reliable, and valid tool to assess the ability of patients to handle their medication. Since especially older patients suffer from multimorbidity and have to take multiple medications, which often also have different dosage forms, it is important to be able to assess not only the ability to self-administer pills, but also oral drops, eye-drops, insulin pens, patches and inhalers, which are often included in the medication regimen of older patients. Up to now, such a tool, which is able to assess the ability to self-administer medication in different dosage forms, is lacking. However, this ability must be recognized and integrated into the medication regimen to ensure treatment success. If patients omit medication, use the wrong dose, or administer their medication in a wrong way, this can lead to over- or undertreatment, which might endanger patients’ health and thus functional state and quality of life. On the one hand, assessment results allow adjusting medication regimens to individual patients’ abilities and needs to ensure independence and hence a better quality of life; on the other hand, insufficient abilities can be compensated for by inclusion of nursing services to secure correct medication administration and thus functional health and independence in other activities of daily living. Thus, the ABLYMED study will help to improve older patients’ quality of life and allocate limited healthcare resources in a reasonable way. In future studies, the assessment tool developed in this study sample will be validated in independent patient samples using pill count and clinical parameters of disease control such as blood pressure, blood glucose, lung capacity, cognitive function etc. as comparative variables to evaluate convergent validity as an important part of construct validity.

## Data Availability

The datasets used and/or analyzed during the current study are available from the corresponding author on reasonable request.

## Abbreviations

BFI-10: Big-Five-Inventory-10
IADL: Instrumental Activities of Daily Living
MedMaIDE: Medication Management Instrument for Deficiencies in the Elderly
MMSE: Mini-mental state examination
MNA-SF: Mini Nutritional Assessment Short Form
ZVT-G: Zahlen-Verbindungs-Test-G (trail making test for gerontopsychological research questions)
